# The Influence of Parental Divorce on Educational Ambitions of 18/19 Year-Old Adolescents from Oslo, Norway

**DOI:** 10.1007/s10826-014-0090-6

**Published:** 2014-12-11

**Authors:** Henok Zeratsion, Cecilie B. Bjertness, Espen Bjertness, Madeleine Dalsklev, Ole R. Haavet, Jon A. Halvorsen, Lars Lien, Bjørgulf Claussen

**Affiliations:** 1 Institute of Health and Society, University of Oslo, P.O. Box 1130, Blindern, 0318 Oslo, Norway; 2 Institute of Psychology, University of Oslo, Oslo, Norway; 3 Department of Dermatology, Oslo University Hospital, Oslo, Norway; 4 National Center for Dual Diagnoses, Innlandet Hospital Trust HF, Ottestad, Norway; 7 Hedmark University College, Elverum, Norway

**Keywords:** Adolescents, Educational ambition, Educational performance, Internalised mental health problems, Parental divorce

## Abstract

Former studies have shown that children and adolescents of divorced parents have significantly poorer educational attainment than their peers from continuously married parents. Educational ambition is important because it has relationship with educational attainment. Our aim was to investigate the associations between parental divorce and educational ambitions among adolescents in the Scandinavian region. Data were used from Young-HUBRO surveys that were conducted in Oslo in the years 2000/2001 and 2004. A change in educational ambition was investigated in a prospective study (n = 1,861) by comparing 18/19 year-olds who experienced late parental divorce with adolescents of continuously married parents. 18/19 year-old adolescents who experienced parental divorce during childhood or adolescence were compared in a cross-sectional study (n = 2,391) with their peers from continuously married parents. Multinomial logistic regression models were fitted to include, among others, mental health problem as a potential confounder. The prospective study showed that a change from ambition for university/college education to having undecided educational ambition was significantly higher among adolescents with experience of late parental divorce than among adolescents of continuously married parents (OR 1.8; 95 % CI 1.1–3.0). In the cross-sectional study, adolescents who experienced parental divorce during childhood or adolescence were more likely to have undecided educational ambition, compared to their peers from continuously married parents (OR 1.3; 95 % CI 1.1–1.8). In conclusion, experience of parental divorce seems to be associated with undecided educational ambition among 18/19 year-old adolescents. Mechanisms that reduce the adverse influence of parental divorce on educational ambitions need to be in place.

## Introduction

Findings about the consequences of parental divorce on social, psychological, and health problems in children and adolescents have not been consistent (Amato and Keith 1991; Biblarz and Gottainer 2000; Breivik and Olweus 2006; Bjørklund et al. 2007; Hemminki and Chen 2006; Steele et al. 2009; Zeratsion et al. 2013). Contradictory findings were also reported about associations between family structure and educational ambitions (Garg et al. 2002, 2007).

Using a large sample of Canadian adolescents, Garg et al. (2007) reported that adolescents from single-parent families had lower educational ambitions than those from two-parent families. Such a difference in ambitions may depend on individual, family and social factors. These factors have been discussed from theoretical perspectives (Amato 1993; Hetherington 1989).

Individual factors that have been mostly studied in relation to a child’s adjustment in the face of parental divorce are age, gender and personality (Hetherington and Stanley-Hagan 1999). Some children possess attributes that increase their vulnerability to adverse effects, while others have better characteristics that foster their resilience in coping with divorce (Amato and Keith 1991).

Younger children may be more adversely affected by parental divorce than adolescents as younger children find it difficult to understand family events, and fear loss of support resources from one or both of the parents (Allison and Furstenberg 1989) but most studies did not find significant effect of child’s age at the time of divorce (Amato 1993). Availability of alternative social support networks that compensate for the lost family resources might be the reason for inconsistent findings about associations between parental divorce and the child’s age.

Gender differential effect of parental divorce was not supported by a meta-analysis (Amato and Keith 1991). Still, boys and girls may differ in their response to parental divorce, especially during middle childhood and adolescence. While girls are more likely to react with “over-controlled” behavior and depression, boys are more likely to develop conduct problems, increase substance use, drop-out from school and experience a decline in academic performance (Rodriguez and Arnold 1998).

In our sample of a cross-sectional study, adolescents with poor mental health reacted to parental divorce with poor adjustment abilities (Zeratsion et al. 2013). The level of social competence, intelligence, temperament and self-esteem contribute to children’s ability to protect themselves from the adverse effects of parental divorce (Hetherington 1989).

Family factors emphasize on parental absence in the household. A family composed of two-biological parents is considered to have an optimal family environment for children (Amato and Keith 1991; Astone and McLenahan 1991; Størksen et al. 2005). Adolescents living in households where one of the biological parents is not present exhibit more adjustment problems and academic difficulties than adolescents living with continuously married parents (Demo and Acock 1996; Fröjd et al. 2011). A study of educational attainment in South-Eastern Norway followed-up 9,749 students after they completed primary education, and found that adolescents living with both biological parents were more likely to complete secondary education than their counterparts living in other form of family structure (Markussen and Sandberg 2005; Markussen et al. 2006). Thus, adolescents of divorced parents may have lower educational ambitions than their peers from intact two-parent families. The argument in this perspective is that each of the biological parents is important resource of emotional support, practical assistance, information and guidance. The amount of resources children lose in relation to parental absence may vary with the factor that led to this absence. Single-parent family due to divorce was reported to have more adverse effect on children’s well-being than single-parent family due to death of a parent (Amato and Keith 1991).

Parental divorce during childhood or early adolescence deprives children mainly of the opportunity to get a male role model (McLanahan 1988), because usually the father leaves the household (Størksen et al. 2005). Father’s absence strongly contributes to the change in parenting practices and family involvement in children’s educational activities (Hetherington et al. 1998; Rodriguez and Arnold 1998). As non-custodial fathers were more likely to maintain contact with sons than with daughters, the link between parental divorce and educational attainment was stronger among female than among male children of divorce (Amato and Keith 1991). The impact of family involvement on academic self-schema of adolescents was stated to be much greater than socioeconomic factors regardless of the marital status of parents. This means that if parents continue to be supportive and actively involved after divorce, there may not be significant change in the adolescent’s orientation to education (Garg et al. 2007).

Social factors may include ethnicity and economic status of a family. Ethnicity is an important social factor because different ethnic groups may react differently to divorce (Amato 1994). A post-divorce single-parent family is expected to be economically less favorable for raising children than intact families (Hetherington et al. 1998; McLanahan 1988). Studies that controlled for the effect of family income have still found significantly more adjustment problems among children of divorce than among children of continuously married parents (Amato and Keith 1991; Demo 1993).

Ambition has been seen as a trait in psychological perspectives, while sociologists consider it to be the product of parental, social or socioeconomic environment. Mentioning the desire to attain ends as its centerpiece, Judge and Kammeyer-Mueller (2012) defined ambition as the persistent and generalized striving for success, attainment, and accomplishment. Emphasized in this definition is that ambition is about attainment rather than achievement.

In investigations of how adolescents vary in their ambitions, there is a need to recognize the social nature of ambition. An individual’s ambition and future attainment are influenced by the social structural positions that the individual inhabit (Baird et al. 2008). Thus, it is important to investigate if ambition is influenced by family structure (Garg et al. 2007).

Educational ambition of adolescents is important because it is associated with educational performance (Garg et al. 2007; Judge and Kammeyer-Mueller 2012; Sewell et al. 1969). Educational performance was significantly poorer among adolescents of divorced parents compared to peers from continuously married parents, as reported in a cross-sectional study in Norway (Lauglo 2008). Adolescents who experienced parental divorce before the age of 14 were found to have lower educational performance than those who lived with continuously married parents, regardless of the stability of the post-divorce family when the adolescent was 14–18 year-old (Sun and Li 2008). With a focus on academic performance, conduct behaviors and amount of joy at school, Størksen et al. (2005) had found that Norwegian adolescents of divorced parents had more school behaviors than their counterparts who did not experience parental divorce.

Contrary to the findings above, the grades attained by students who experienced parental divorce did not have significant difference from the grades of those without such experience (Kaye 1989). According to this prospective longitudinal study, parental divorce was associated with educational outcomes of both gender groups only in the short-term; long-term effects on test scores were observed only among boys.

Findings from a cross-sectional study of educational attainment in Norway showed that parental divorce experienced before the offspring turned 16 years of age, influence a child’s educational transition from compulsory school to lower secondary, from lower to higher secondary and from higher secondary to bachelor’s degree level (Steele et al. 2009). In the same study this association was found to get weaker with increase in the child’s age at the time of family disruption. In their longitudinal and cross-sectional studies of a large sample from Sweden, Jonsson and Gåhler (1997) found that children who experienced parental divorce were less likely to continue to or reach upper secondary school, compared to their peers from intact families. A comparison of single-parent families also found that children raised by widowed single mothers were more likely to continue education beyond high school compared to children raised by divorced mothers (Biblarz and Gottainer 2000). This indicates that the adverse effect of parental divorce is greater even than death of a parent.

Parental divorce is known to be one of the main causes of single-parent or stepparent family. Hence, the consequences of parental divorce have also been explained by referring to effects of these family structures. Adolescents in single-parent families or stepfamilies received less encouragement and less help with school work than their peers who lived with both biological parents (Astone and McLenahan 1991).

The paucity of studies about educational ambitions of children of divorced parents in their late adolescence was the rationale for the present study. The aim was to investigate the relationship of parental divorce with educational ambitions among 18/19 year-old adolescents. We hypothesized that compared to adolescents of continuously married parents the educational ambition of 18/19 year-old adolescents is significantly reduced, or changed to undecided ambition after experiencing parental divorce during the 3 years preceding the age of 18/19 years. This was investigated using a prospective study design. The second hypothesis was that 18/19 year-old adolescents with experience of parental divorce during childhood or adolescence have lower ambitions for education or have undecided educational ambitions when compared to their peers from continuously married parents. A cross-sectional study was conducted to test this hypothesis.

## Method

### Participants

This was a cohort study where all 15/16 year-old, students in primary school in the city of Oslo (n = 4,273) were invited to participate in the baseline survey of Young-HUBRO in their school-year 2000/2001. The participants (n = 3,811) were followed-up 3 years later. We excluded those who stated that parents’ marital status was other than married/cohabitants or divorced/separated (n = 222) and those who gave contradictory answers in the two surveys (n = 55). We had a new dataset for baseline Young-HUBRO (n = 3,534) of which 1,143 individuals did not respond to the question on marital status. This gave a sample of 2,391 adolescents for the cross-sectional study. In the prospective study, since our focus was on late parental divorce, we excluded 530 adolescents who had experienced parental divorce before the age of 15/16 years, giving a sample of 1,861 adolescents. Informed consent was given by students and parents. The study was approved by the Regional Ethical Committee for Medical Research.

### Procedure

In the Young-HUBRO Health Study in Oslo, self-administered questionnaires were mostly answered in the class room sessions. At follow-up, 13 % of the participants received the questionnaire by post because they did not attend school. A project assistant was present at the schools to inform the students about the survey and to administer data collection. Both the baseline and follow-up surveys were conducted at the end of the school-year. Additional description of the sample is available in another article (Bjertness et al. 2010).

### Measures

A four-page questionnaire was used to collect data both at baseline and follow-up. Data contained in the questionnaire were used to define the explanatory and outcome variables.

#### Explanatory Variables

Parental marital status dichotomized into “continuously married” and “divorced” was our main independent variable. Continuously married was defined as adolescents who had never experienced parental divorce until the second survey in 2004. “Late divorce” referred to parental divorce or separation that occurred when the offspring was between age 15/16 and 18/19. “Divorce” was defined as all parental divorces that occurred before the follow-up.

Potential confounders were identified based on evidence from analysis of bivariate associations (Table 1) and previous literature. Ethnicity was dichotomized into “western” and “non-western” based on parents’ place of birth.

**Table 1:** Independent variables across parental marital status in a prospective and a cross-sectional study of 18/19 year-old adolescents in Oslo, in 2004 (percentages)

Independent variables	Prospective study (N = 1,861)			Cross-sectional study (N = 2,391)		
	All	Continuously married	Late divorce	All	Continuously married	Divorced
Gender						
Male	838 (45.0)	793 (45.3)	45 (41.3)	1,069 (44.7)	793 (45.3)	276 (43.2)
Female	1,023 (55.0)	959 (54.7)	64 (58.7)	1,322 (55.3)	959 (54.7)	363 (56.8)
Ethnicity						
Western	1,608 (86.7)	1,515 (86.7)	93 (85.3)	2,104 (88.4)	1,515 (86.8)	589 (92.6)**
Non-western	246 (13.3)	230 (13.3)	16 (14.7)	277 (11.6)	230 (13.2)	47 (7.4)
Family economy						
Average and below	420 (23.0)	381 (22.1)	39 (36.4)**	646 (27.4)	381 (22.1)	265 (41.8)**
Above average	1,408 (77.0)	1,340 (77.9)	68 (63.6)	1,709 (72.6)	1,340 (77.9)	369 (58.2)
Social support						
Low	157 (8.5)	149 (8.6)	8 (7.3)	210 (8.8)	149 (8.6)	61 (9.5)
High	1,692 (91.5)	1,591 (91.4)	101 (92.7)	2,169 (91.2)	1,591 (91.4)	578 (90.5)
Mother’s educational level						
Higher secondary school or above	1,191 (66.9)	1,132 (67.5)	59 (56.7)*	1,551 (67.4)	1,132 (67.5)	419 (67.0)
Below higher secondary school	589 (33.1)	544 (32.5)	45 (43.3)	750 (32.6)	544 (32.5)	206 (33.0)
Father’s educational level						
Higher secondary schools or above	1,284 (72.2)	1,216 (72.3)	68 (70.1)	1,641 (71.8)	1,216 (72.3)	425 (70.5)
Below higher secondary school	495 (27.8)	466 (27.7)	29 (29.9)	644 (28.2)	466 (27.7)	178 (29.5)
Internalized mental health problems						
No mental health problems	1,565 (84.1)	1,482 (84.6)	83 (76.1)*	1,985 (83.0)	1,482 (84.6)	503 (78.7)**
Have mental health problems	296 (15.9)	270 (15.4)	26 (23.9)	406 (17.0)	270 (15.4)	136 (21.3)
Educ. performance at primary school						
Poor	188 (10.6)	173 (10.3)	15 (14.4)	273 (12.0)	173 (10.3)	100 (16.4)**
Average	765 (43.1)	729 (43.6)	36 (34.6)	974 (42.7)	729 (43.6)	245 (40.1)
Outstanding	823 (46.3)	770 (46.1)	53 (51.0)	1,036 (45.3)	770 (46.1)	266 (43.5)
Educ. ambition at primary school						
University/college	1,180 (64.0)	1,111 (64.0)	69 (63.9)			
Secondary education	384 (20.8)	360 (20.7)	24 (22.2)			
Undecided	281 (15.2)	266 (15.3)	15 (13.9)			

Significant association at ** *p* < 0.01 or * *p* < 0.05

Family economy was dichotomized into “above average” and “average and below”, based on the statement “I believe, relative to others in Norway, my family has: (1) ‘poor economy’, (2) ‘average economy’; (3) ‘good economy’; or (4) ‘very good economy’”.

Social support was created by summarizing the response of two questions that focused on availability of help: “How many persons outside your immediate family are so close to you that you can rely on to get help (1) if you have personal problems, (2) if you have practical problems (for example, school assignments)?” Those who answered “0” or “1” for each of these two separate questions formed the low social support category and the rest were grouped to form the high social support category. This gave a cut-off point at 85th and 78th percentile of the sample in the first and the second question, respectively. The mean inter-item correlation value was 0.42, and this value falls within an optimal range for a scale with fewer than 10 items (Briggs and Cheek 1986). We chose this proxy measure of social support because the number of people to whom the adolescents can talk and rely on for help when they have personal or practical problems has also been used in other studies (Schraedley et al. 1999). Furthermore, perceived social support during adolescence was found to be associated with academic achievement as the extended network of family members and peers provide a broad base of security that enables the student to overcome challenges of school environment (Levitt et al. 1994).

Mother’s educational level and father’s educational level were two separate categorical variables obtained by linking the Young-HUBRO dataset to a database at Statistics Norway with individual personal data from all schools and universities in Norway. Using the Norwegian Standard for Classification of Education (Statistics Norway, 2000), that classified educational levels into nine categories, we have dichotomized each of these variables into “higher secondary school or above” and “below higher secondary school.”

Internalized mental health problems were measured using the test Hopkin’s Symptoms Check List (HSCL-10). The ten questions were asked to measure the level of anxiety and depression symptoms during the week that preceded the survey day. The items were rated on a scale from 1 (no symptom) to 4 (much of the symptom). An average score for all 10-items of 1.85 was used as cut-off point to dichotomize the variable into “No internal mental health problem” and “Have internal mental health problem”; the second category was formed of scores greater than or equal to 1.85 (Strand et al. 2003). This cut-off point was used in other studies as a valid predictor of mental distress among adolescents (Lien et al. 2007). Reliability was high (Cronbach’s alpha = 0.88).

Educational performance at primary school was based on the question “What was the grade you obtained in your transcript last time in (a) mathematics, (b) written Norwegian, (c) English, (d) social science?” Based on the Norwegian grading system for basic education where 6 was the best grade, we first classified grades 1–2, 3–4, and 5–6 respectively as ‘poor’ (0), ‘average’ (1), and ‘outstanding’ (2) performance in each of the four subjects. Educational performance at primary school was first computed to summarize performance in the four subjects in the form of an index of scores that range from 0 to 8. Then, scores 0–3, 4–5, and 6–8 were respectively used to form the categories ‘poor’, ‘average’, and ‘outstanding’ for this variable.

#### Outcome Variable: Ambition for Education

This variable was based on the question “What is the highest level of education you plan to take?” Seven alternative plans were provided: (1) university or college education of higher level; (2) university or college education of middle level; (3) secondary general education/financial administration; (4) secondary vocational education; (5) 1 year of secondary education; (6) other educational ambitions; (7) undecided. We reduced the categories to three, of which “ambition for university/college education” consisted of the first two alternatives, “ambition for secondary education” referred to alternatives 3, 4, and 5, and “undecided ambition” was alternative 7. Those answering alternative number 6 were excluded.

#### Missing Values

In the sample for the cross-sectional study, only adolescents who participated in both surveys and answered the questions on parental marital status were included. This gave 32 % missing values of the 3,534 respondents in the first Young-HUBRO survey. Non-response was 27 % among western and 42 % among non-western adolescents, and was random at the other independent variables.

### Analysis

Bivariate associations between parental marital status and each of the other independent variables were studied using Pearson Chi square test (Table 1). Multinomial logistic regression models were fitted in both the prospective (Table 2) and the cross-sectional (Table 3) studies. Potential confounders included gender, ethnicity, family economy, social support, mother’s educational level, father’s educational level, internalized mental health problems, and educational performance at primary school. In the prospective design, educational ambition at primary school was also controlled for, making the study an investigation of a change in educational ambition. Change in educational ambition measures the likelihood of making a shift, during the time interval 2000/2001 to 2004, from having ambition for university/college education to having ambition for secondary education or to undecided ambition by comparing adolescents of divorced parents with adolescents of continuously married parents.

**Table 2:** Change in educational ambition, from year 2000/2001 to 2004, among adolescents who experienced late parental divorce (n = 109) versus the change among their peers whose parents were continuously married (n = 1,752), in a prospective study of 18/19 year-old adolescents in Oslo (N = 1,861), after adjustment in multinomial logistic regressions for educational ambition and other potential confounders measured in 2000/2001

Independent variables	Ambition for secondary education	Undecided educational ambition
Crude results		
Late parental divorce (ref = continuously married)	2.0 (0.9–4.0)	1.7 (1.1–2.7)*
Adjusted results		
Late parental divorce (ref = continuously married)	1.2 (0.4–3.2)	1.8 (1.1–3.0)*
Gender (ref = boys)	0.4 (0.2–0.7)**	0.9 (0.7–1.3)
Ethnicity (ref = western)	1.1 (0.5–2.2)	0.5 (0.3–0.8)
Family economy (ref = average and below)	0.5 (0.3–0.9)	0.7 (0.5–0.9)*
Social support (ref = low)	1.7 (0.6–4.7)	0.8 (0.5–1.2)
Mother’s education (ref = higher sec. sch. or above)	1.7 (0.9–2.9)	0.9 (0.7–1.3)
Father education (ref = higher sec. sch. or above)	1.3 (0.7–2.2)	1.1 (0.8–1.5)
Internalized problems (ref = no)	1.3 (0.7–2.8)	1.0 (0.7–1.5)
Educ. performance at primary sch. (ref = outstanding)		
Poor educ. performance	3.7 (1.6–8.4)**	1.5 (0.9–2.6)
Average educ. performance	1.6 (0.8–3.2)	1.3 (0.9–1.7)
Educ. ambition at primary sch. (ref = uni./college educ.)		
Secondary education	11.0 (6.0–20.6)**	3.6 (2.5–5.1)**
Undecided	2.3 (0.9–5.8)	2.9 (2.0–4.1)**

Ambition for university/college education was the reference category. Odds ratio (95 % confidence interval)

Significant association ** *p* < 0.01 or * *p* < 0.05

**Table 3:** Educational ambitions among adolescents who experienced parental divorce during childhood or adolescence (n = 639) compared to ambitions among those whose parents were continuously married (n = 1,752) in a cross-sectional study of 18/19 year-old adolescents in Oslo (N = 2,391) after adjustment in multinomial logistic regressions for potential confounders measured at age 15/16

Independent variables	Ambition for secondary education	Undecided educational ambition
Crude results		
Parental divorce (ref = continuously married)	1.9 (1.3–2.7)**	1.6 (1.2–1.9)**
Adjusted results		
Parental divorce (ref = continuously married)	1.5 (0.9–2.3)	1.3 (1.1–1.8)
Gender (ref = boys)	0.5 (0.3–0.8)*	1.1 (0.8–1.3)
Ethnicity (ref = western)	0.7 (0.4–1.4)	0.4 (0.3–0.7)**
Family economy (ref = average and below)	0.6 (0.4–0.9)	0.8 (0.6–1.0)
Social support (ref = low)	1.3 (0.6–2.8)*	0.7 (0.5–1.1)
Mother’s education (ref = higher sec. sch. or above)	1.6 (1.0–2.5)*	1.0 (0.7–1.3)
Father education (ref = higher sec. sch. or above)	1.6 (1.0–2.5)	1.1 (0.9–1.5)
Internalized problems (ref = no)	1.4 (0.8–2.5)	1.0 (0.7–1.4)
Educ. performance at primary sch. (ref = outstanding)		
Poor educ. performance	14.6 (7.5–28.3)**	3.3 (2.2–4.9)**
Average educ. performance	3.8 (2.1–6.8)*	2.0 (1.5–2.6)*

Ambition for university/college education was the reference category. Odds ratios (95 % confidence interval)

Significant association ** *p* < 0.01 or * *p* < 0.05

## Results

Pearson Chi square (χ^2^) was used to investigate binary associations. Late parental divorce that occurred when the adolescents were 15/16 to 18/19 year-old had a statistically significant association with family economy (χ^2^ = 11.7; *df* = 1; *p* value <0.005), with mother’s education level (χ^2^ = 5.2; *df* = 1; *p* < 0.023), and with internalized mental health problems (χ^2^ = 5.5; *df* = 1; *p* < 0.019) (Table 1). Experience of parental divorce during childhood and adolescence had significant association with ethnicity (χ^2^ = 15.2; *df* = 1; *p* < 0.005), family economy (χ^2^ = 89.9; *df* = 1; *p* < 0.005), internalized mental health problems (χ^2^ = 11.5; *df* = 1; *p* < 0.005), and educational performance (χ^2^ = 15.5; *df* = 2; *p* < 0.005) at primary school.

Odds ratio (OR) was used as a measure of association between the explanatory variables and educational ambition. The degrees of freedom was equal to 1 for all reported regression results. Our prospective study shows that compared to adolescents of continuously married parents, those who experienced late parental divorce were more likely to change to undecided ambition rather than to have ambition for university/college education (OR 1.7; *p* = 0.03). The same was found even after the model was controlled for potential confounders (OR 1.8; *p* = 0.03) (Table 2).

Girls compared with boys were less likely to change from ambition for university/college education to ambition for secondary education in their transition from primary to secondary education (OR 0.4; *p* < 0.01). In such transition, adolescents with above average family income had less chance of changing from ambition for university/college to undecided ambition than those in the lower family income group (OR 0.7; *p* = 0.02). In addition, the change from ambition for university/college to secondary education was more common among 18/19 year-old adolescents with poor rather than with outstanding educational performance at age 15/16 (OR 3.7; *p* < 0.01). 18/19 year-olds who had ambition for secondary education when they were in primary school were more likely to have maintained that ambition (OR 11.0; *p* < 0.01), or changed to undecided ambition (OR 3.6; *p* < 0.01) from ambition for university/college.

Crude OR from the cross-sectional study showed that adolescents who experienced parental divorce before 18/19 years of age were more likely to have undecided ambition (OR 1.6; *p* < 0.01), or ambition for secondary education (OR 1.9; *p* < 0.01) rather than to have ambition for university/college education (Table 3). The association between experience of parental divorce and ambition for secondary education turned to be non-significant after the model was adjusted for potential confounders. The adjustment for adolescents’ educational performance at age 15/16 changed this association from significant to non-significant. Girls (OR 0.5; *p* < 0.01) or adolescents from families with above average income (OR 0.6; *p* = 0.03) were less likely to have ambition for secondary education rather than ambition for university/college. Adolescents with poor educational performance at primary school (OR 14.6; *p* < 0.01), or those born to parents with lower educational level (OR 1.6; *p* = 0.03) more often had ambition for secondary education than ambition for university/college at their 18/19 years of age.

Undecided educational ambition was less common among adolescents with non-western ethnic background than among those with western ethnic background (OR 0.4; *p* < 0.01). There was also higher chance for having undecided educational ambition at 18/19 years of age among adolescents who had poor or average educational performance at primary school (OR 3.3; *p* < 0.01).

## Discussion

We found significant change from ambition for university/college education to having undecided ambition among adolescents who experienced late parental divorce than among their counterparts from continuously married parents. The same was found for adolescents who experienced parental divorce any time before 18/19 years of age.

Thus, the hypothesis of reduced ambitions after experience of late parental divorce has been supported by our findings in the prospective study. Similarly, the findings of our cross-sectional study support the hypothesis that 18/19 year-olds who experience parental divorce during childhood or adolescence are more likely to have undecided educational ambition than their peers from continuously married parents.

Although there is scarcity of previous studies on educational ambition, the findings of the present study are consistent with the findings of studies that investigated the influence of parental divorce on future education (Jonsson and Gåhler 1997; Steele et al. 2009). A previous cross-sectional study from Norway showed that experience of parental divorce influence adolescents’ transition from compulsory education all the way up to college level, although the influence was found to get weaker with the adolescents’ age at time of divorce (Steele et al. 2009). Utilizing both prospective and cross-sectional analyses of a large dataset from Sweden, Jonsson and Gåhler (1997) found that adolescents who lived with continuously married parents were more likely to continue at school than those who experienced parental divorce. Deterioration in the household’s social class and educational resources due to moving out of the parent with higher educational and/or income levels was stated to explain the negative effects of parental divorce on a child’s ambition to continue education.

Ambition is important for success in future education (Markussen et al. 2006; Judge and Kammeyer-Mueller 2012) which is influenced by parental divorce (Steele et al. 2009). Family structure influenced the years of schooling in a study of children and adolescents in Sweden and the USA; non-intact family structure variables were negatively associated with the decision to continue education (Bjørklund et al. 2007). A follow-up study from Norway found that the likelihood of dropping out of secondary education was significantly higher among adolescents who lived with a single-parent than their counterparts from two-parent families (Markussen and Sandberg 2005; Markussen et al. 2006). Family structure variables are important because adolescents who live with single parents or stepparents get less encouragement and help with school work than children living with continuously married biological parents (Astone and McLenahan 1991). These explanations are consistent with the theoretical perspective of parental absence (Amato and Keith 1991; Hetherington et al. 1998). Since usually fathers move out from the household (Størksen et al. 2005), father absence is likely to be associated with the deterioration of the educational resources and encouragement that the children of divorced parents encounter. Besides losing the opportunity to get a male role model in the household (McLanahan 1988), children of divorced parents get a reduced parental attention and supervision due to a reduced contact between the offspring and the non-custodial parent (Rodriguez and Arnold 1998). Thus, diminished parenting following parental divorce is a factor that contributes to differences between children of continuously married and divorced parents (Cherlin et al. 1991).

The focus of the present study being investigation of the influence of parental divorce on educational ambition among adolescents, more research needs to be done on the mechanisms of how this influence operates. A previous study found that both boys and girls with mental depression in their 14–16 years of age were at increased risk of significantly poorer educational underachievement in their late adolescence compared to adolescents who did not have mental health symptoms in their middle adolescence (Fergusson and Woodward 2002). The relationship between mental health and parental divorce was investigated in our previous cross-sectional study among 15/16 year-old adolescents where we found significant association (Zeratsion et al. 2013). The findings of these two studies imply the possibility of a mechanism where parental divorce exerts its influence on educational ambition through the association it has with mental health. However, no evidence of significant association between mental health symptoms and educational ambition was found in our present study.

Looking closely into the effect of non-intact families, it can be noted that the same type of family structure resulting from different causes of parental absence may not necessarily lead to the same educational outcomes. Children from divorced single-mother families were found to have significantly lower educational levels than children from widowed single-mother families (Biblarz and Gottainer 2000). Thus, not only single-mother family was important but also how the father became absent from the household.

Educational ambitions were found to be negatively associated with cigarette smoking (Hashim et al. 2009) and drug use (Newcomb et al. 1989) in adolescence. These risk behaviors that are more common among adolescents of divorced parents than among same-aged peers from continuously married parents (Zeratsion et al. under review), may increase the chance of having undecided ambition among adolescents of divorced parents.

As strength of the present study data was used from a prospective follow-up of a population with 89 % participation at first survey and a 68 % follow-up rate 3 years later. We were able to conduct a prospectively longitudinal study and a cross-sectional study. While the cross-sectional study was used to compare groups on a specific time, the prospectively longitudinal study enabled us to more accurately estimate the influence of a change in parental marital status—from married to divorced—on educational ambition when the adolescents were between 15/16 and 18/19 year-olds. Although loss-to-follow-up was 32 % in the present study, association measures are shown to be robust to such levels of loss-to-follow-up (Bjertness et al. 2010). Non-response was more common among non-western adolescents making our estimates to be more unsecure for ethnic non-western group. Otherwise, no significant differences were found between missing cases and responders. Having the opportunity to link our dataset with a related database in Statistics Norway, we were able to use in our analyses sufficiently measured potential confounders.

This study was not without limitations. The use of more potential confounders including risk behaviors and measures of parental conflict would have improved model specification. Since we do not have multi-age cohort it was not possible to examine the influence of parental divorce on various age groups. The low incidence rate of parental divorce during a follow-up period of 3 years resulted in a relatively small sub-sample of those with experience of late parental divorce. This low incidence rate might have been the reason for being unable to discern significant associations between experience of parental divorce and change from ambition for university/college education to secondary education although the odds ratio was 1.20.
